# Final Analysis of Post‐Marketing Surveillance for Avelumab + Axitinib in Patients With Renal Cell Carcinoma in Japan

**DOI:** 10.1002/cam4.70275

**Published:** 2025-01-21

**Authors:** Norio Nonomura, Taito Ito, Masashi Sato, Makiko Morita, Mie Ogi, Masahiro Kajita, Mototsugu Oya

**Affiliations:** ^1^ Department of Urology Osaka University Graduate School of Medicine Osaka Japan; ^2^ Medical Department Merck Biopharma Co., Ltd., Tokyo, Japan, an Affiliate of Merck KGaA Darmstadt Germany; ^3^ Research and Development Merck Biopharma Co., Ltd., Tokyo, Japan, an Affiliate of Merck KGaA Darmstadt Germany; ^4^ Global Patient Safety Japan Merck Biopharma Co., Ltd., Tokyo, Japan, an Affiliate of Merck KGaA Darmstadt Germany; ^5^ Global Development Operations Merck Biopharma Co., Ltd., Tokyo, Japan, an Affiliate of Merck KGaA Darmstadt Germany; ^6^ Department of Urology Keio University School of Medicine Tokyo Japan

**Keywords:** avelumab, effectiveness, post‐marketing surveillance, renal cell carcinoma, safety

## Abstract

**Introduction:**

Avelumab, an anti‐programmed death ligand 1 antibody, was approved in combination with axitinib for curatively unresectable or metastatic renal cell carcinoma (RCC) in Japan in December 2019. Because the pivotal JAVELIN Renal 101 study included a limited number of Japanese patients, post‐marketing surveillance (PMS) was required to evaluate outcomes (safety and effectiveness) in patients with RCC who received avelumab + axitinib treatment in clinical practice in Japan.

**Materials and Methods:**

We report data from prospective, noncomparative, multicenter, observational PMS in patients with RCC who received ≥ 1 dose of avelumab. Patients were enrolled between December 2019 (date of regulatory approval) and May 2021. The primary objective was to evaluate safety, defined as adverse drug reactions (ADRs) of safety specifications occurring during an observation period of ≤ 52 weeks for each patient. The secondary objective was to evaluate effectiveness, including best overall response and overall survival (OS).

**Results:**

In total, 328 patients were included in the safety and effectiveness analysis sets. Overall, 173 patients (52.7%) had ADRs of safety specifications of any grade, most commonly thyroid dysfunction (*n* = 69 [21.0%]), infusion reaction (*n* = 65 [19.8%]), and hepatic disorders (*n* = 45 [13.7%]). Objective responses occurred in 118 patients (36.0%), including complete or partial responses in 13 (4.0%) and 105 (32.0%), respectively; the disease control rate was 75.6%. The 12‐month OS rate was 83.7% (95% CI, 78.9%–87.4%).

**Discussion:**

This PMS confirmed the safety, tolerability, and effectiveness of avelumab + axitinib in patients with RCC in clinical practice in Japan, with a benefit–risk profile comparable to that observed in clinical trials.

## Graphical abstract

We report data from prospective, noncomparative, multicenter, observational post‐marketing surveillance in patients with curatively unresectable or metastatic renal cell carcinoma who received avelumab + axitinib in Japan between December 2019 and May 2021 (*N* = 328). Adverse drug reactions of safety specifications of any grade occurred in 52.7% of patients, disease control rate was 75.6%, and the 12‐month OS rate was 83.7%. Our results confirmed the safety, tolerability, and effectiveness of avelumab + axitinib in patients with renal cell carcinoma in clinical practice in Japan, with a benefit–risk profile comparable to that observed in clinical trials.

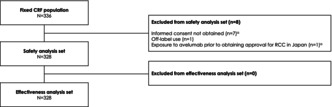

## 1 Introduction

Avelumab is a monoclonal antibody that inhibits programmed death ligand 1 (PD‐L1), and axitinib is an inhibitor of receptor tyrosine kinases, including vascular endothelial growth factor receptor (VEGFR)‐1, VEGFR‐2, and VEGFR‐3 [1, 2, 3]. Avelumab in combination with axitinib has been approved in various countries worldwide for first‐line treatment of patients with advanced renal cell carcinoma (RCC) [1, 2]. In addition, avelumab monotherapy has been approved worldwide for treatment of metastatic Merkel cell carcinoma and for first‐line maintenance treatment of advanced urothelial carcinoma in patients without disease progression following platinum‐based chemotherapy [1, 2].

In Japan, avelumab was the first PD‐L1 inhibitor to be approved following its approval for the treatment of curatively unresectable Merkel cell carcinoma in 2017 [4]. In addition, avelumab + axitinib was approved for the treatment of curatively unresectable or metastatic RCC in December 2019 [5], and avelumab monotherapy was approved for maintenance treatment following chemotherapy in curatively unresectable urothelial carcinoma in February 2021 [6].

The approval of avelumab + axitinib for patients with RCC in Japan was based on the results of the Phase 3 JAVELIN Renal 101 study (NCT02684006), which included a subgroup of Japanese patients [7, 8]. In the overall study population, avelumab + axitinib significantly improved progression‐free survival (PFS) compared with sunitinib [8]. After extended follow‐up (≥ 28 months in all patients), median PFS was 13.9 versus 8.5 months (hazard ratio [HR], 0.67 [95% CI, 0.568–0.785]; *p* < 0.0001) and median overall survival (OS) was not reached versus 37.8 months (HR, 0.79 [95% CI, 0.643–0.969]; *p* = 0.0116; the analysis of OS remained immature at the time of the publication) [9]. The objective response rate (ORR) with avelumab + axitinib versus sunitinib was 59.3% versus 31.8%, including complete response (CR) in 4.8% versus 3.2% of patients, respectively [9]. The safety profile of avelumab + axitinib in combination was consistent with that observed in previous monotherapy studies [8]. Avelumab + axitinib also demonstrated a positive benefit‐to‐risk ratio versus sunitinib across all International Metastatic Renal Cell Carcinoma Database Consortium (IMDC) risk groups and in patients with PD‐L1‐positive tumors [8, 9]. In the subgroup analysis of patients enrolled in JAVELIN Renal 101 in Japan (*n* = 67) who received avelumab + axitinib or sunitinib, median PFS was 16.6 versus 11.2 months (HR, 0.66 [95% CI, 0.296–1.464]), median OS was not reached in either arm (HR, 0.53 [95% CI, 0.042–6.693]), and the ORR was 60.6% versus 17.6%, respectively [7]. Based on these findings, the Japanese Urological Association clinical practice guidelines for RCC (updated in 2022) recommend avelumab + axitinib for first‐line treatment of patients with low‐, intermediate‐, and high‐risk clear cell RCC as first‐line therapy [10].

Because of the limited number of Japanese patients who were enrolled in the JAVELIN Renal 101 study, post‐marketing surveillance (PMS) was required to further evaluate the safety and effectiveness of avelumab + axitinib in patients with RCC in general clinical practice in Japan. Here, we report final PMS data.

## 2 Materials and Methods

### 2.1 Study Design and Patient Population

Prospective, noncomparative, observational PMS was conducted at multiple centers throughout Japan to evaluate the safety and effectiveness of avelumab + axitinib in patients with RCC. The PMS population included patients with curatively unresectable or metastatic RCC in Japan who started treatment with avelumab + axitinib and were enrolled in the PMS from December 20, 2019 (date of regulatory approval) to May 31, 2021. Because the PMS was designed as all‐case surveillance, all patients with RCC who received ≥ 1 dose of avelumab were included. Patient enrollment was centralized. Investigators completed electronic or paper case report forms (CRFs) and data were collected and analyzed by the sponsor.

### 2.2 Endpoints

The primary objective was to evaluate the safety of avelumab + axitinib in the PMS population. Data on adverse drug reactions (ADRs) of safety specifications, that is, noxious and unintended effects that were deemed to have a causal relationship with avelumab, were collected. ADRs of safety specifications were defined by a Japanese Risk Management Plan and were recorded in the case report form (CRF). ADRs of safety specifications included the following: interstitial lung disease, pancreatitis, hepatic function disorders, colitis or severe diarrhea, thyroid dysfunction, adrenal insufficiency, pituitary disorders, Type 1 diabetes mellitus, myocarditis, nerve disorders (including Guillain–Barré syndrome), renal disorders, myositis/rhabdomyolysis, infusion reaction, encephalitis/meningitis, and myasthenia gravis. Grading was determined by investigators with reference to Common Terminology Criteria for Adverse Events version 5.0.

The secondary objective was to evaluate the effectiveness of avelumab + axitinib in the PMS population. Effectiveness endpoints were best overall response and OS. Best overall response (i.e., CR, partial response [PR], stable disease [SD], progressive disease [PD], or not evaluable [NE]) was determined by investigators with reference to Response Evaluation Criteria in Solid Tumors (RECIST) version 1.1 [11]. ORR (best response of CR or PR) and disease control rate (DCR; best response of CR, PR, or SD) were calculated. OS was defined as time from start of treatment to death from any cause.

### 2.3 Statistical Analyses

Safety and effectiveness analyses included data from the finalized CRF population, whose CRFs had been collected and data recorded from the start of PMS (December 20, 2019) until database lock (March 22, 2023). The safety analysis population consisted of all patients who received ≥ 1 dose of avelumab treatment, excluding patients who received off‐label treatment (i.e., not administered for curatively unresectable or metastatic RCC), and patients who received avelumab treatment before the approval of avelumab in Japan, or who did not provide consent. The effectiveness analysis population consisted of patients in the safety analysis population, excluding patients who transferred to another hospital and had their information linked from before the transfer.

Safety data were aggregated by system organ class and preferred term, presented as overall frequency and percentages, in addition to worst grade and time of onset. For best overall response, proportions of patients with CR, PR, SD, PD, or response NE were calculated. For ORR and DCR, proportions of patients with 95% confidence intervals (CI) were calculated. OS was estimated using the Kaplan–Meier method, including median, rates at time points, and 95% CIs. Patients who were alive or lost to follow‐up at the end of the observation period were censored at the last observation date.

## 3 Results

### 3.1 Patients and Treatment

**FIGURE 1:**
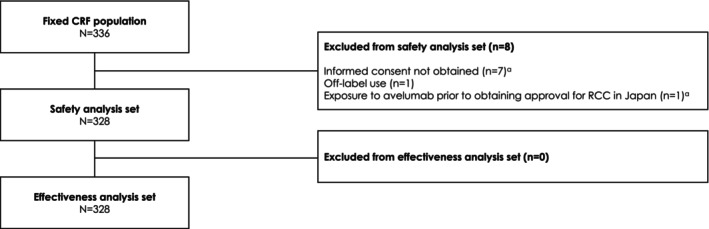
Study flowchart. ^a^One patient met criteria for both “Informed consent not obtained” and “Exposure to avelumab prior to obtaining approval for RCC in Japan.” CRF, case report form; RCC, renal cell carcinoma.

At database lock (March 22, 2023), CRFs had been collected from 336 patients at 160 institutions. After excluding patients who did not provide consent (*n* = 7), received off‐label treatment (*n* = 1), or received avelumab before the approval of avelumab for RCC in Japan (*n* = 1; this patient also did not provide consent), 328 patients were included in safety and effectiveness analysis populations (Figure 1).

At baseline, median age was 70.0 years (range, 12–89 years), and 237 (72.3%) and 91 (27.7%) patients were male and female, respectively (Table 1). ECOG PS was 0‐1 in 300 (91.5%) and ≥ 2 in 26 (7.9%). The majority of patients (*n* = 273 [83.2%]) had Stage IV disease, and a minority (*n* = 29 [8.8%]) had Stage III disease. IMDC risk classification was favorable in 90 patients (27.4%), intermediate with one risk factor in 91 (27.7%), intermediate with two risk factors in 58 (17.7%), and poor in 60 (18.3%); baseline characteristics by IMDC risk classification are shown in Table S1. Before receiving avelumab + axitinib treatment, 212 patients (64.6%) had undergone surgery, 31 (9.5%) had received radiation therapy, and 39 (11.9%) had received drug treatment. No pregnant or breastfeeding patients were enrolled.

**TABLE 1:** Baseline characteristics.

Characteristic	*N* = 328
Age	
Median (range), years	70.0 (12–89)
< 65 years, *n* (%)	100 (30.5)
≥ 65 to < 75 years, *n* (%)	130 (39.6)
≥ 75 years, *n* (%)	98 (29.9)
Sex, *n* (%)	
Male	237 (72.3)
Female	91 (27.7)
Weight, median (range), kg	60.4 (29.5–113.9)
ECOG PS, *n* (%)	
0	208 (63.4)
1	92 (28.0)
≥ 2	26 (7.9)
Unknown	2 (0.6)
Disease stage classification, *n* (%)	
III	29 (8.8)
IV	273 (83.2)
Other	4 (1.2)
Unknown	22 (6.7)
Metastatic disease, *n* (%)	
Yes	308 (93.9)
No	20 (6.1)
Pathologic classification, *n* (%)	
Clear cell carcinoma	271 (82.6)
Non‐clear cell carcinoma	22 (6.7)
Other	35 (10.7)
IMDC risk classification, *n* (%)	
Favorable	90 (27.4)
Intermediate (one risk factor)	91 (27.7)
Intermediate (two risk factors)	58 (17.7)
Poor	60 (18.3)
Unknown	29 (8.8)
Comorbidity, *n* (%)	
Renal impairment	88 (26.8)
Hepatic impairment	14 (4.3)
Interstitial lung disease	1 (0.3)
Autoimmune disease	6 (1.8)
Prior treatment, *n* (%)	
Surgery	212 (64.6)
Radiation	31 (9.5)

Abbreviation: IMDC, International Metastatic Renal Cell Carcinoma Database Consortium.

The median duration of treatment was 7.8 months with avelumab and 7.1 months with axitinib, and relative median dose intensity was 100% (range, 20.0%–101.0%) and 80.0% (range, 20.0%–182.5%), respectively (Table S2). At data cutoff, 116 patients (35.4%) remained on avelumab and/or axitinib treatment and 212 (64.6%) had discontinued treatment. The most common reasons for discontinuation were disease progression (32.1% [68 of 212 patients]) and ADRs of safety specifications (27.4% [58 of 212 patients]). After discontinuation, 74 patients received subsequent treatment, most commonly cabozantinib (*n* = 48) and nivolumab (*n* = 20) (Table S3).

### 3.2 Safety

Overall, 173 patients (52.7%) had an ADR of safety specifications; the highest grade was Grade 3 in 43 (13.1%) and Grade 4 in 13 (4.0%) (Table 2). Two patients had an ADR leading to death: interstitial lung disease (*n* = 1) and Guillain–Barré syndrome (*n* = 1). The most common ADRs of safety specifications (those occurring in > 10% of patients) were thyroid dysfunction (69 [21.0%]), infusion reaction (65 [19.8%]), and hepatic function disorders (45 [13.7%]). ADRs of safety specifications of any grade occurred in 127 of 228 patients aged ≥ 65 years (55.7%), 44 of 88 patients with renal impairment (50%), and 7 of 14 patients with hepatic impairment (50%).

**TABLE 2:** Adverse drug reactions of safety specifications.

	*N* = 328	
Overview of ADRs of safety specifications, *n* (%)		
Any grade	173 (52.7)	
Grade ≥ 3	56 (17.1)	
Leading to death (Grade 5)	2 (0.6)	
Leading to discontinuation of avelumab	60 (18.3)	
Requiring high‐dose steroid treatment^a^	24 (7.3)	
Due to infusion reaction^b^	12 (3.7)	
Due to ADRs of safety specifications (other than infusion reaction)^b^	13 (4.0)	
ADRs of safety specifications, *n* (%)	Any grade	Grade 3/4
Thyroid dysfunction	69 (21.0)	6 (1.8)
Infusion reaction	65 (19.8)	7 (2.1)
Hepatic function disorders	45 (13.7)	17 (5.2)
Nerve disorders (including Guillain–Barré syndrome)	14 (4.3)	6 (1.8)
Adrenal insufficiency	13 (4.0)	8 (2.4)
Interstitial lung disease	10 (3.1)	3 (0.9)
Colitis or severe diarrhea	8 (2.4)	8 (2.4)
Renal disorders	6 (1.8)	1 (0.3)
Type 1 diabetes mellitus	3 (0.9)	2 (0.6)
Pituitary disorders	2 (0.6)	1 (0.3)
Pancreatitis	1 (0.3)	1 (0.3)
Myocarditis	1 (0.3)	1 (0.3)

Abbreviation: ADR, adverse drug reaction.

^a^ ≥ 40 mg total daily dose of prednisone or equivalent.

^b^ One patient had both infusion reaction and an ADR of safety specifications (other than infusion reaction).

**FIGURE 2:**
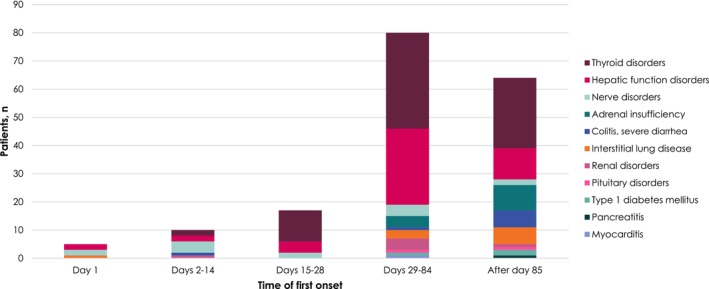
Time of the first onset of adverse drug reactions of safety specifications (excluding infusion reaction).

The time of onset of ADRs of safety specifications was Day 1 in 59 patients (18.0%; infusion reaction in 56 patients), between Days 2 and 14 in 12 patients (3.7%), between Days 15 and 28 in 18 patients (5.5%), between Days 29 and 84 in 69 patients (21.0%), and Day ≥ 85 in 58 patients (17.7%) (Figure 2). High‐dose steroids were administered to 24 patients (7.3%), which was for infusion reactions in 12 (3.7%) and ADRs of safety specifications (other than infusion reactions) in 13 (4.0%) (1 patient was included in both categories). High‐dose steroids were administered for hepatic function disorders in 6 patients; interstitial lung disease and nerve disorders in 2 patients each; and colitis, myocarditis, and pancreatitis in 1 patient each. By the end of the observation period, 107 patients (32.6%) had recovered from an ADR, 61 (18.6%) had an ADR that had improved, 3 (0.9%) had recovered from an ADR with sequelae, and 32 (9.8%) had not recovered from an ADR (patients with > 1 ADR are counted in all relevant categories).

#### 3.2.1 Infusion Reactions

**FIGURE 3:**
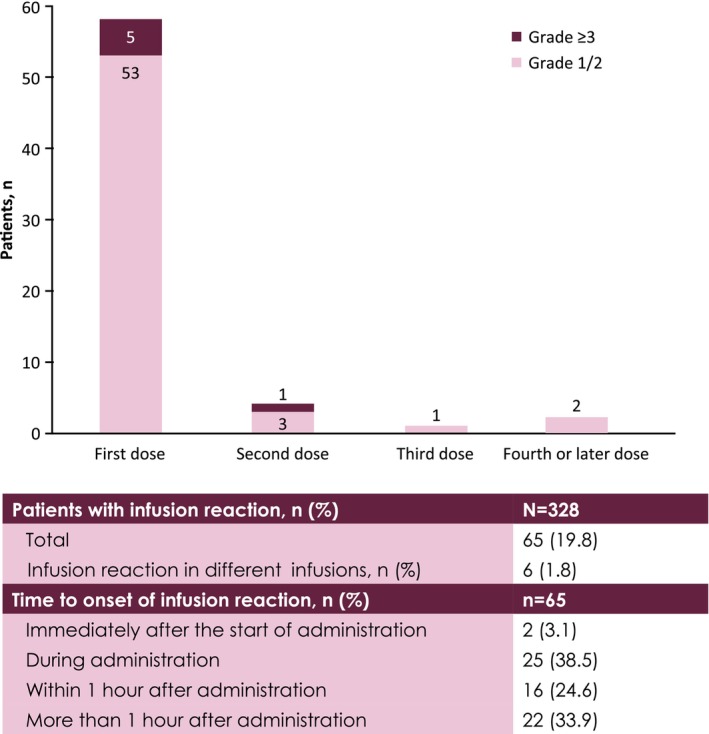
First onset of infusion reaction by grade.

In total, 65 patients (19.8%) had an ADR of safety specifications that was classified as an infusion reaction of any grade. Seven patients (2.1%) had grade ≥ 3 infusion reactions. No fatal infusion reactions occurred. Among the 65 patients who had an infusion reaction of any grade, most had onset at the first dose (*n* = 58 [89.2%]), with a small proportion having onset on the first day of the second dose (*n* = 4 [6.2%]) or later doses (*n* = 3 [4.6%]) (Figure 3). Among the patients who had an infusion reaction, most reactions occurred immediately after the start of avelumab administration, during administration, or within 1 h after administration (*n* = 43 [66.2%]). Six patients had > 1 infusion reaction. Of the 65 patients with infusion reactions, 64 had recovered or improved by the end of the observation period. Among all 328 patients, 323 (98.5%) received premedication for infusion reactions at the time of the first avelumab administration. The most common premedication regimens were acetaminophen + diphenhydramine (*n* = 228 [69.5%]) and acetaminophen + chlorpheniramine maleate (*n* = 50 [15.2%]). Among the 323 patients who received premedication, infusion reaction occurred at the first dose of avelumab in 57 patients (17.6%) (Table S4).

#### 3.2.2 Other ADRs of Safety Specifications

Incidences of other ADRs of safety specifications were as follows: 21.0% (*n* = 69) for thyroid dysfunction, 13.7% (*n* = 45) for hepatic function disorders, 4.3% (*n* = 14) for nerve disorders (including Guillain–Barré syndrome), 4.0% (*n* = 13) for adrenal insufficiency, 3.1% (*n* = 10) for interstitial lung disease, 2.4% (*n* = 8) for colitis or severe diarrhea, 1.8% (*n* = 6) for renal disorders, 0.9% (*n* = 3) for Type 1 diabetes mellitus, 0.6% (*n* = 2) for pituitary disorders, and 0.3% (*n* = 1) each for pancreatitis and myocarditis. The most common grade ≥ 3 ADRs of safety specifications were hepatic function disorders in 17 patients (5.2%), colitis or severe diarrhea in 8 (2.4%), and adrenal insufficiency in 8 (2.4%) (Table 2). The median time to onset of the most common ADRs of safety specifications of any grade was 50.0 days (range, 4–310 days) for thyroid dysfunction and 46.0 days (range, 1–295 days) for hepatic function disorders (Figure S1).

### 3.3 Effectiveness

**FIGURE 4:**
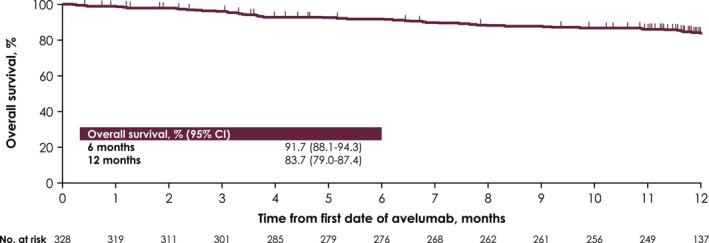
Kaplan–Meier curve of overall survival in the effectiveness analysis set.

**FIGURE 5:**
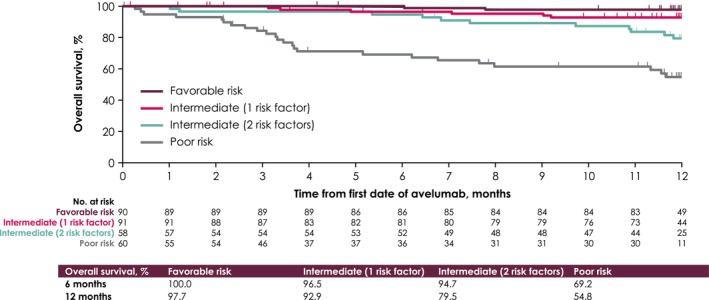
Kaplan–Meier curve of overall survival by International Metastatic Renal Cell Carcinoma Database Consortium risk category.

Among 328 patients in the effectiveness analysis set, best overall response was CR in 13 patients (4.0%) and PR in 105 patients (32.0%), and response was NE in 2 patients (0.6%) and not reported in 33 patients (10.1%), resulting in an ORR of 36.0% (95% CI, 30.8%–41.4%). In addition, 130 patients (39.6%) had SD as best response, resulting in a DCR of 75.6% (95% CI, 70.6%–80.2%) (Table 3). Median OS was not reached, and 6‐ and 12‐month OS rates were 91.7% (95% CI, 88.1%–94.3%) and 83.7% (95% CI, 79.0%–87.4%), respectively (Figure 4). Median OS was also not reached in any IMDC risk group, and 6‐ and 12‐month OS rates were 100.0% and 97.7% in the favorable risk group, 96.5% and 92.9% in the intermediate‐risk/1 risk factor group, 94.7% and 79.5% in the intermediate‐risk/two risk factors group, and 69.2% and 54.8% in the poor‐risk group (Figure 5).

**TABLE 3:** Best overall response.

Parameter	*N* = 328
Best overall response, *n* (%)	
Complete response	13 (4.0)
Partial response	105 (32.0)
Stable disease	130 (39.6)
Progressive disease	45 (13.7)
Not evaluable	2 (0.6)
Not reported	33 (10.1)
Objective response rate, *n* (%) [95% CI]	118 (36.0) [30.8–41.4]
Disease control rate, *n* (%) [95% CI]	248 (75.6) [70.6–80.2]

## 4 Discussion

This PMS analysis provides the first comprehensive dataset of patients with curatively unresectable or metastatic RCC treated with avelumab + axitinib combination therapy in general clinical practice in Japan. The reported data show that the safety and effectiveness of avelumab + axitinib in this population is comparable to observations in clinical trials. ADRs were manageable, and rates of ADRs and time to onset were as expected, confirming the acceptable tolerability of avelumab + axitinib. The PMS population had some differences in demographic and disease characteristics compared with the avelumab + axitinib arm of the phase 3 JAVELIN Renal 101 trial, including higher proportions of patients aged ≥ 65 years (PMS vs. JAVELIN Renal 101, 70% vs. 39%) or aged ≥ 75 years (30% vs. 7%), or with no prior surgery/nephrectomy (35% vs. 20%), and inclusion of subgroups with ECOG PS ≥ 2 (7.9%), non‐clear cell RCC (6.7%), Stage III disease (8.8%), or renal impairment (26.8%) [8, 12]. However, proportions of patients in different IMDC risk groups were generally similar (PMS vs. JAVELIN Renal 101, favorable in 27% vs. 21%, intermediate in 45% vs. 61%, and poor in 18% vs. 16%) [8].

The safety profile of avelumab + axitinib in the PMS population was comparable to observations in clinical trials. Any‐grade or Grade ≥ 3 ADRs of safety specifications occurred in 53% and 17% in this PMS analysis, respectively, with similar rates of ADRs of safety specifications seen in older patients and those with renal or hepatic impairment compared with the overall population. Types of ADRs were similar to those observed in the JAVELIN Renal 101 trial [8]. In the PMS population, the most common ADRs of safety specifications were thyroid dysfunction (21%), infusion reaction (20%), and hepatic disorders (14%), comparable to rates observed in a reanalysis of JAVELIN Renal 101 data for ADRs of safety specifications reported in the Japanese Risk Management Plan for avelumab (32%, 26%, and 20%, respectively) [6]. ADRs were also manageable, with high‐dose steroids administered to 7.3% of patients overall, and 10% of patients having ADRs that had not recovered or improved by the end of the observation period. In 65 patients who had an infusion reaction, most occurred at administration of the first dose of avelumab (89%) and within 1 h of administration (66%). According to the prescribing information for avelumab in Japan, it is recommended that patients receive premedication with antihistamines or antipyretic analgesics to mitigate the risk of infusion reactions [5]. In the PMS population, 98% of patients received premedication, indicating strong adherence with this requirement in general clinical practice.

The ORR in the PMS population was 36%, which was lower than the ORR of 51% observed in the initial analysis of JAVELIN Renal 101 [8]. This may be due to differences in patient and disease characteristics known to be associated with worse outcomes, such as older age, worse ECOG PS, no prior surgery, and non‐clear cell pathologic classification [12, 13, 14, 15], in addition to other differences between the populations. In the JAVELIN Renal 101 trial, the odds ratio for ORR was numerically higher in patients with prior nephrectomy versus without prior nephrectomy in the avelumab + axitinib arm [16]; hence, the ORR may have been affected by the high proportion of patients without prior surgery in general clinical practice. Assessment of tumor shrinkage and RECIST response may also be performed less often in general clinical practice than in clinical trials, and tumor diameter may not be measured rigorously in early assessments because of the potential for pseudoprogression. However, in the PMS and JAVELIN Renal 101 populations, DCRs (76% vs. 81%) and proportions of patients with PD as best response (14% vs. 12%) were similar [8], suggesting that similar assessments of disease progression were performed in general clinical practice.

In the PMS population, median OS was not reached by the data cutoff, and the 12‐month OS rate was 84%. Similarly, in JAVELIN Renal 101, median OS was not reached at the third interim analysis [9], and the 12‐month OS rate was 86% [17]. Differences in OS between IMDC risk groups were consistent with observations in JAVELIN Renal 101, including the shorter OS in the poor‐risk group versus other groups [9]. Of note, the poor‐risk group in the PMS population included a markedly higher proportion of patients with ECOG PS ≥ 2 versus the other risk groups (28% vs. 0%–7%). However, 12‐month OS rates were similar between patients in intermediate‐risk (one risk factor) and favorable risk groups, which may be due to the similarities between these groups in general clinical practice.

Data obtained from PMS have some limitations. First, source data from CRFs were not verified using medical records, per GPSP ordinance. Second, methods of assessment in this PMS were different than in the JAVELIN Renal 101 study, and data were not reviewed by an independent data monitoring committee; thus, comparisons with JAVELIN Renal 101 data must be interpreted with caution. Third, the PMS was noncomparative and observational and did not collect information on patients who did not receive avelumab + axitinib; therefore, it is not possible to confirm that observations were due to drug exposure.

In conclusion, data from PMS confirm the acceptable safety and tolerability of avelumab + axitinib in patients with curatively unresectable or metastatic RCC in general clinical practice in Japan. Effectiveness was generally consistent with observations in the avelumab + axitinib arm of the Phase 3 JAVELIN Renal 101 study. These results indicate that the positive benefit–risk profile of avelumab + axitinib in general clinical practice in Japan is comparable to findings from clinical trials.

## Author Contributions

**Norio Nonomura:** conceptualization (equal), investigation (equal), supervision (equal), writing – review and editing (equal). **Taito Ito:** conceptualization (equal), methodology (equal), project administration (equal), writing – review and editing (equal). **Masashi Sato:** data curation (equal), formal analysis (equal), methodology (equal), writing – review and editing (equal). **Makiko Morita:** validation (equal), writing – review and editing (equal). **Mie Ogi:** data curation (equal), writing – review and editing (equal). **Masahiro Kajita:** conceptualization (equal), visualization (equal), writing – original draft (equal), writing – review and editing (equal). **Mototsugu Oya:** conceptualization (equal), investigation (equal), supervision (equal), writing – review and editing (equal).

## Ethics Statement

This PMS was conducted in accordance with Japanese regulations for Good Post‐Marketing Study Practice (GPSP) [18]. The protocol was reviewed by all participating institutions. The approval of the ethical committee/institutional review board and informed consent was obtained from patients based on requirements of each institution.

## Conflicts of Interest

Norio Nonomura reports receipt of royalties from Shionogi, receipt of honoraria from Astellas, AstraZeneca, Janssen, Merck Biopharma Co., Ltd., Tokyo, Japan, an affiliate of Merck KGaA, Darmstadt, Germany, and Takeda, receipt of research funds from Astellas, IQVIA, Parexel, and TOSOH, and receipt of scholarship endowments from Nippon Shinyaku, ONO, Pfizer, TAIHO, Takeda, and Yakult. Taito Ito, Masashi Sato, Makiko Morita, Mie Ogi, and Masahiro Kajita report employment with Merck Biopharma Co., Ltd., Tokyo, Japan, an affiliate of Merck KGaA, Darmstadt, Germany. Mototsugu Oya reports receipt of honoraria from Bayer, Bristol Myers Squibb, Eisai, Merck Biopharma Co., Ltd., Tokyo, Japan, an affiliate of Merck KGaA, Darmstadt, Germany, Merck & Co., Kenilworth, NJ, ONO, Pfizer, and Takeda, receipt of manuscript fees from Pfizer Japan, and receipt of scholarship endowments from Bayer, Eisai, ONO, and Takeda.

## Supporting information


Data S1.

## Data Availability

Any requests for data by qualified scientific and medical researchers for legitimate research purposes will be subject to the healthcare business of Merck KGaA, Darmstadt, Germany's Data Sharing Policy. All requests should be submitted in writing to the healthcare business of Merck KGaA, Darmstadt, Germany's data sharing portal (https://www.emdgroup.com/en/research/our‐approach‐to‐research‐and‐development/healthcare/clinical‐trials/commitment‐responsible‐data‐sharing.html). When the healthcare business of Merck KGaA, Darmstadt, Germany has a co‐research, co‐development, or co‐marketing or co‐promotion agreement, or when the product has been out‐licensed, the responsibility for disclosure might be dependent on the agreement between parties. Under these circumstances, the healthcare business of Merck KGaA, Darmstadt, Germany will endeavor to gain agreement to share data in response to requests.
