# Supplementary material for: Final Analysis of Post‐Marketing Surveillance for Avelumab + Axitinib in Patients With Renal Cell Carcinoma in Japan
Source: Cancer Med. 2025 Jan 21;14(2):e70275. doi: 10.1002/cam4.70275 (PMC11750683; doi:10.1002/cam4.70275)

**Supporting** **Information**

**Table S1.** Baseline characteristics by International Metastatic Renal Cell Carcinoma Database Consortium risk classification.

|  | **Favorable  (n=90)** | **Intermediate, 1 risk factor (n=91)** | **Intermediate, 2 risk factors (n=58)** | **Poor  (n=60)** |
| --- | --- | --- | --- | --- |
| Sex, n (%) |  |  |  |  |
| Male | 62 (68.9) | 64 (70.3) | 41 (70.7) | 47 (78.3) |
| Female | 28 (31.1) | 27 (29.7) | 17 (29.3) | 13 (21.7) |
| Age |  |  |  |  |
| Median (range), years | 68 (37-88) | 71 (12-89) | 71.5 (29-89) | 68.5 (28-88) |
| ≥65 years, n (%) | 58 (64.4) | 68 (74.7) | 42 (72.4) | 37 (61.7) |
| ≥75 years, n (%) | 16 (17.8) | 36 (39.6) | 20 (34.5) | 16 (26.7) |
| Weight, median (range), kg | 64.8 (33.0-113.9) | 61.3 (29.5-100.1) | 59.7 (34.3-97.0) | 56.5 (39.4-102.9) |
| ECOG PS, n (%) |  |  |  |  |
| 0 | 79 (87.8) | 64 (70.3) | 35 (60.3) | 16 (26.7) |
| 1 | 11 (12.2) | 25 (27.5) | 19 (32.8) | 27 (45.0) |
| ≥2 | 0 | 2 (2.2) | 4 (6.9) | 17 (28.3) |
| Disease stage, n (%) |  |  |  |  |
| III | 9 (10.0) | 8 (8.8) | 5 (8.6) | 3 (5.0) |
| IV | 70 (77.8) | 75 (82.4) | 52 (89.7) | 55 (91.7) |
| Other/not recorded | 2 (2.2) | 1 (1.1) | 0 | 0 |
| Metastatic lesion, n (%) |  |  |  |  |
| Yes | 86 (95.6) | 88 (96.7) | 53 (91.4) | 56 (93.3) |
| No | 4 (4.4) | 3 (3.3) | 5 (8.6) | 4 (6.7) |
| Pathologic classification, n (%) |  |  |  |  |
| Clear cell | 83 (92.2) | 74 (81.3) | 46 (79.3) | 45 (75.0) |
| Non-clear cell | 6 (6.7) | 10 (11.0) | 3 (5.2) | 2 (3.3) |
| Other | 1 (1.1) | 7 (7.7) | 9 (15.5) | 13 (21.7) |
| Comorbidity, n (%) |  |  |  |  |
| Renal impairment | 23 (25.6) | 27 (29.7) | 20 (34.5) | 12 (20.0) |
| Hepatic impairment | 4 (4.4) | 5 (5.5) | 0 | 4 (6.7) |
| Interstitial lung disease | 0 | 0 | 1 (1.7) | 0 |
| Autoimmune disease | 1 (1.1) | 4 (4.4) | 0 | 1 (1.7) |
| Other | 27 (30.0) | 24 (26.4) | 18 (31.0) | 17 (28.3) |
| Prior treatment, n (%) |  |  |  |  |
| Surgery | 84 (93.3) | 60 (65.9) | 30 (51.7) | 22 (36.7) |
| Radiation | 7 (7.8) | 6 (6.6) | 5 (8.6) | 9 (15.0) |
| Drug treatment | 10 (11.1) | 10 (11.0) | 5 (8.6) | 9 (15.0) |

**Table S2.** Treatment characteristics and reasons for discontinuation.

|  | **N=328** | |
| --- | --- | --- |
|  | **Avelumab** | **Axitinib** |
| Received treatment, n (%) | 328 (100) | 327 (99.7) |
| Duration of treatment, median, months | 7.8 | 7.1 |
| Relative dose intensity, median (range), % | 100.0 (20.0-101.0) | 80.0 (20.0-182.5) |
| Still receiving treatment at data cutoff, n (%) | 116 (35.4) | |
| Discontinued treatment, n (%) | 212 (64.6) | |
| Progression of underlying disease | 68 (32.1) | |
| ADRs of safety specifications | 58 (27.4) | |
| Surgery | 18 (8.5) | |
| Patient preference | 16 (7.5) | |
| Death | 13 (6.1) | |
| Transfer to different hospital | 10 (4.7) | |
| Other reason | 33 (15.6) | |

Patients with >1 reason for discontinuation are included in all relevant rows.

ADR, adverse drug reaction.

**Table S3.** Drugs received as subsequent treatment as monotherapy or in combination after discontinuation of avelumab + axitinib.

|  | **N=328** | **Reason for discontinuation** | |
| --- | --- | --- | --- |
|  |  | **Progression of underlying disease n=68** | **ADRs of safety specifications n=58** |
| Any subsequent treatment, n (%)^a^ | 74 (22.7) | 48 (70.6) | 21 (36.2) |
| Cabozantinib | 48 (14.6) | 37 (54.4) | 9 (15.5) |
| Nivolumab | 20 (6.1) | 9 (13.2) | 7 (12.1) |
| Pazopanib | 4 (1.2) | 1 (1.5) | 3 (5.2) |
| Pembrolizumab | 3 (0.9) | 2 (2.9) | 1 (1.7) |
| Investigational agent | 2 (0.6) | 1 (1.5) | 1 (1.7) |
| Sorafenib | 2 (0.6) | 1 (1.5) | 1 (1.7) |
| Sunitinib | 2 (0.6) | 2 (2.9) | 0 |
| Everolimus | 1 (0.3) | 0 | 1 (1.7) |
| Pembrolizumab + axitinib | 1 (0.3) | 0 | 1 (1.7) |
| Temsirolimus | 1 (0.3) | 1 (1.5) | 0 |

ADR, adverse drug reaction.

^a^ Patients with >1 subsequent treatment are included in all relevant rows.

**Table S4.** Premedication received at first dose in the safety analysis set (N=328).

|  | **n (%)** | **Proportion with infusion reaction, n/N (%)** |
| --- | --- | --- |
| Received premedication | 323 (98.5) | 57/323 (17.6) |
| Acetaminophen + diphenhydramine | 228 (69.5) | 37/228 (16.2) |
| Acetaminophen + chlorpheniramine | 50 (15.2) | 14/50 (28.0) |
| Other | 45 (13.7) | 6/45 (13.3) |
| No premedication | 5 (1.5) | 1/5 (20.0) |

**Figure S1.** Time to onset of adverse drug reactions of safety specifications. IQR, interquartile range.


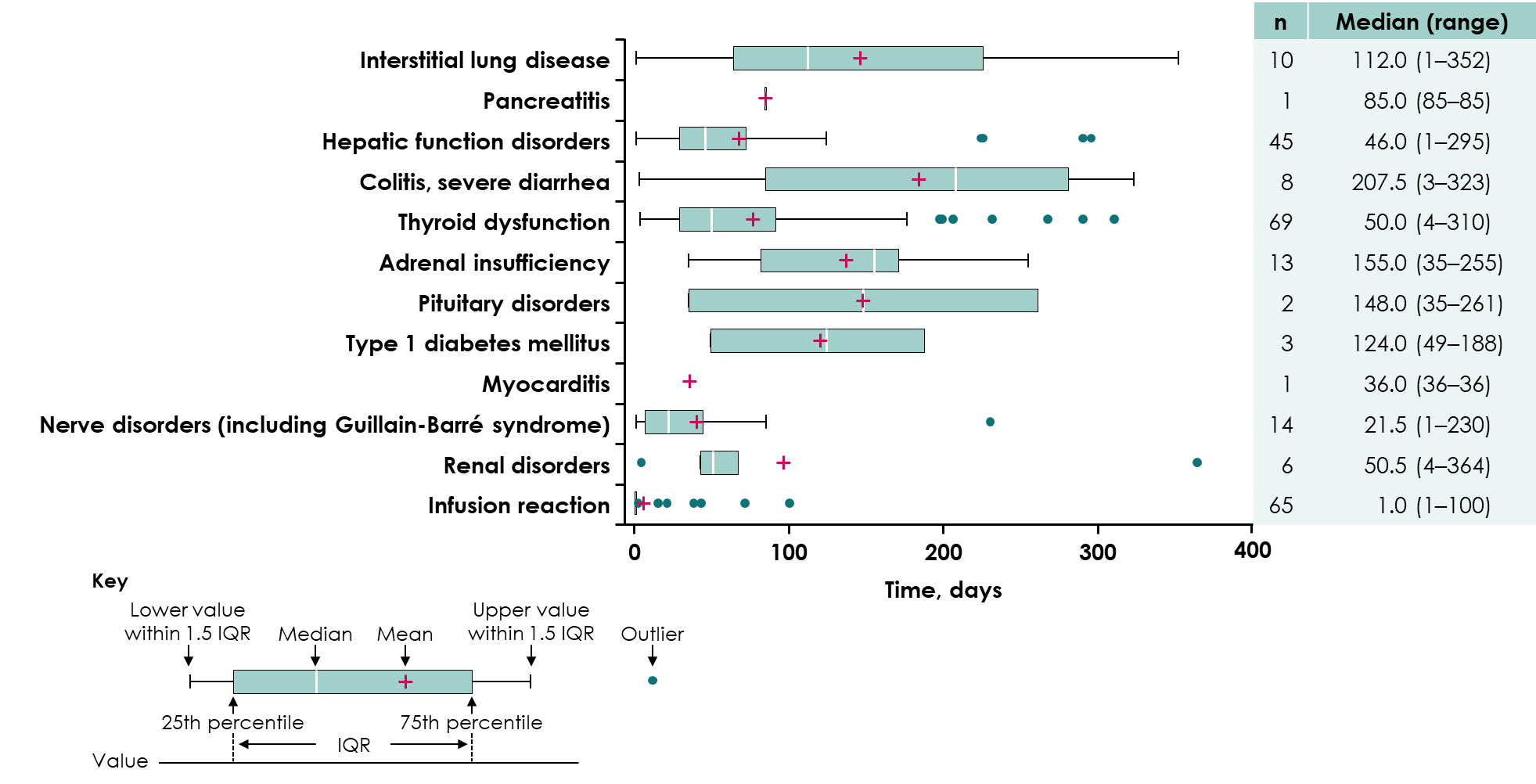

Supplement: Supplementary file 1 — Data S1. [file CAM4-14-e70275-s001.zip › Supporting Information_RCC_Japan_PMS_Manuscript_02Jan24.docx]
